# γ-Oryzanol Improves Cognitive Function and Modulates Hippocampal Proteome in Mice

**DOI:** 10.3390/nu11040753

**Published:** 2019-03-31

**Authors:** Wiramon Rungratanawanich, Giovanna Cenini, Andrea Mastinu, Marc Sylvester, Anne Wilkening, Giulia Abate, Sara Anna Bonini, Francesca Aria, Mariagrazia Marziano, Giuseppina Maccarinelli, Maurizio Memo, Wolfgang Voos, Daniela Uberti

**Affiliations:** 1 Department of Molecular and Translational Medicine, University of Brescia, 25123 Brescia, Italy; wiramon.r@gmail.com (W.R.); andrea.mastinu@unibs.it (A.M.); g.abate001@unibs.it (G.A.); sara.bonini@unibs.it (S.A.B.); f.aria@unibs.it (F.A.); m.marziano@unibs.it (M.M.); giuseppina.maccarinelli@unibs.it (G.M.); maurizio.memo@unibs.it (M.M.); 2 Institut für Biochemie und Molekularbiologie, Universität Bonn, 53115 Bonn, Germany; gcenini@gmail.com (G.C.); msylvest@uni-bonn.de (M.S.); annewilkening@gmx.de (A.W.); wolfgang.voos@uni-bonn.de (W.V.)

**Keywords:** γ-oryzanol, cognitive function, brain, hippocampus, behavior, antioxidant, proteomics, *Oryza sativa*

## Abstract

Rice (*Oryza sativa* L.) is the richest source of γ-oryzanol, a compound endowed with antioxidant and anti-inflammatory properties. γ-Oryzanol has been demonstrated to cross the blood-brain barrier in intact form and exert beneficial effects on brain function. This study aimed to clarify the effects of γ-oryzanol in the hippocampus in terms of cognitive function and protein expression. Adult mice were administered with γ-oryzanol 100 mg/kg or vehicle (control) once a day for 21 consecutive days following which cognitive behavior and hippocampal proteome were investigated. Cognitive tests using novel object recognition and Y-maze showed that long-term consumption of γ-oryzanol improves cognitive function in mice. To investigate the hippocampal proteome modulated by γ-oryzanol, 2D-difference gel electrophoresis (2D-DIGE) was performed. Interestingly, we found that γ-oryzanol modulates quantitative changes of proteins involved in synaptic plasticity and neuronal trafficking, neuroprotection and antioxidant activity, and mitochondria and energy metabolism. These findings suggested γ-oryzanol as a natural compound able to maintain and reinforce brain function. Although more intensive studies are needed, we propose γ-oryzanol as a putative dietary phytochemical for preserving brain reserve, the ability to tolerate age-related changes, thereby preventing clinical symptoms or signs of neurodegenerative diseases.

## 1. Introduction

Rice is the main grain in the human diet worldwide and represents the cultural identity and unity of many countries [1,2]. In the genus *Oryza*, there are about 24 species of rice containing more than 100,000 varieties. Among these, *Oryza sativa* and *Oryza glaberrima* are the main species consumed globally [2,3,4]. Rice has an important value not only as a staple food to supply a large fraction of energy needs, but also promoting and maintaining a healthy status owing to its bioactive nutrients [5]. Rice contains essential amino acids and dietary fibers, and is rich in various bioactive phytochemicals such as vitamin E (tocopherols and tocotrienols), γ-aminobutyric acid (GABA), anthocyanins, phenolics, flavones, and γ-oryzanol [6,7]. As compared to other plant-based diets, rice is the richest source of γ-oryzanol. The name γ-oryzanol is derived from rice as it was first discovered in rice bran oil (“oryza-”) and consists of a hydroxyl group (“-nol”), thus giving the name “γ-oryzanol” [8]. γ-Oryzanol was first extracted in 1954 from rice bran oil and was initially considered as a single compound [8]. Later, the molecular structure of γ-oryzanol was revealed as a mixture of phytosteryl ferulates containing ferulic acid esters of phytosterols (sterols and triterpene alcohols) [9]. In γ-oryzanol, there are at least 10 phytosteryl ferulates that have been characterized. Among these, the major components accounting for approximately 80% are cycloartenyl, 24-methylenecycloartanyl, campesteryl, and sitosteryl ferulates [9,10]. 

γ-Oryzanol has been proven safe for consumption with no major side effect [11,12]. Numerous studies have reported health-beneficial properties of γ-oryzanol including anti-hyperlipidemic, anti-ulcerogenic, anti-carcinogenic, anti-inflammatory, anti-diabetic, and antioxidant effects [10,11]. We recently demonstrated the mechanism by which γ-oryzanol regulates cellular redox homeostasis [13]. In particular, by using an in vitro model, we found that γ-oryzanol activates nuclear factor erythroid 2-related factor 2 (Nrf2) pathway inducing Nrf2 nuclear translocation and activation of Nrf2-ARE phase II enzymes [14]. γ-Oryzanol is found to cross the blood–brain barrier in intact form and to be distributed highly in the brain [15,16]. Studies of γ-oryzanol in the brain have illustrated its role in modulating the function of the hypothalamus and anterior pituitary gland [17,18,19]. Recently, in a rat model of streptozotocin-induced sporadic Alzheimer’s disease (AD), γ-oryzanol was also reported to preserve memory and cognitive function [20]. These findings suggested an additional potential of γ-oryzanol in the brain. However, the effects of γ-oryzanol in hippocampus, a crucial region of the brain responsible for learning and memory processing and cognition [21,22], has not yet been fully elucidated. Therefore, this study aimed to clarify the effects of long-term treatment of γ-oryzanol in the hippocampus. To this end, we engaged novel object recognition (NOR) and Y-maze tests to evaluate cognitive performance [23,24], and 2D-difference gel electrophoresis (2D-DIGE) to investigate the alteration of hippocampal protein expression from mice administered with γ-oryzanol compared with vehicle (control) for 3 consecutive weeks.

## 2. Materials and Methods 

### 2.1. Animals

Male B6/129PF2 mice 12–14 months old, *n* = 20, average weight 43 g, were purchased from The Jackson Laboratories, Bar Harbor, ME, USA. Mice were housed in a 12 h light/dark cycle, 3–4 per cage and maintained at 20–22 °C with 50% relative humidity. They were ad libitum given water and normal weighted chow (3.95 kcal/g of standard diet 4RF21, Mucedola: carbohydrate 70%, protein 20% and fat 10%). All experiments were conducted under the European Communities Council Directive of 1986 (86/609/EEC) and approved by the Italian Ministry of Health and the Animal Care and Use Committee of the University of Brescia.

### 2.2. Dose Calculation

Oral dosage of γ-oryzanol in mice was calculated as 100 mg/kg according to the dose translation formula from human to animal based on the body surface area of the daily dosage of γ-oryzanol 500 mg/day in human [25,26].

### 2.3. Chemicals and Treatment

Mice were divided into 2 groups:(1) γ-oryzanol group received γ-oryzanol (Sigma-Aldrich) 100 mg/kg by oral gavage once a day for 3 weeks (*n* = 10); (2) control group received vehicle (normal saline) by oral gavage once a day for 3 weeks (*n* = 10). After 3 weeks, behavioral tests were performed. Mice were subjected to sequentially behavioral battery tests: first rotarod for locomotor activity, then Y-maze for spatial memory, and NOR for long-term memory. In particular, mice were placed and habituated to the behavioral room for 30 min at the beginning of each test, and all the tests were conducted by the same experimenters. Then, mice were sacrificed and their hippocampi were isolated. The hippocampi were then homogenized in ice-cold buffer (0.32 M Sucrose, 1 M Tris/HCl pH 8, 0.1 mM MgCl_2_, 0.1 mM ethylenediaminetetraacetic acid, 10 μg/mL leupeptin, 10 μg/mL pepstatin, 10 μg/mL aprotinin) and sonicated for 1 min on ice. The samples were then stored at −80 °C until further analysis.

### 2.4. Rotarod Test

Prior to the cognitive tests, rotarod was used to evaluate motor performance. Mice (n_γ-oryzanol_ = 10, n_control_ = 10) were placed one by one on the rotarod treadmill (Ugo Basile) to perform a trial of 30 s at the constant speed of 2 rpm. Then, the test was performed immediately at an initial intensity of 2 rpm and a final intensity of 20 rpm for 300 s. The time that each mouse spent on the top of the rotarod treadmill was recorded.

### 2.5. Y-Maze

Y-maze paradigm was used to measure the working memory ability of mice. The black Y-maze consisted of 3 identical arms placed at 120°. Each arm had height 15 cm, width 8 cm, and length 30 cm. Mice (n_γ-oryzanol_=10, n_control_ = 10) were placed at the end of one arm and allowed to move freely through Y-maze for 5 min during the test. The number of arm entries was video-recorded, tracked, and automatically analyzed by EthoVision XT software (Noldus). Max alternation was defined as the number of possible alternations counted by the total number of arms entered minus 2. Alternation was defined as consecutive entries into 3 different arms and counted only if a mouse entered into the 3 arms of maze (without revisiting the first arm at the third visit) as reported by Holter et al., 2015 [27].

### 2.6. Novel Object Recognition (NOR)

NOR assay was used to explore cognitive performance in the long-term memory. This protocol foresaw 3 consecutive days as reported by Antunes and Biala, 2012 and Leger et al., 2013 [28,29] in a 40 × 40 cm Plexiglas square arena. On Day 1, the mice (n_γ-oryzanol_ = 10, n_control_ = 10) were habituated to an open field arena for 5 min. On Day 2, the mice were exposed to 2 identical objects for a 5-min period. On Day 3, one of the objects was replaced with a new object, and the mice were let to explore the arena for 5 min. Each trial was recorded and the videos were automatically analyzed by EthoVision XT software (Noldus). The time spent with the familiar and novel objects was measured. In addition, discrimination index calculated as [(time novel − time familiar)/(time novel + time familiar) × 100%] was also evaluated.

### 2.7. Two-Dimensional Electrophoresis

Two-dimensional (2D) electrophoresis was performed as previously described [30]. After the behavioral tests, mice were sacrificed and their hippocampi were taken to perform 2D-DIGE. 9 hippocampi per group were used. For each group, 3 hippocampi were pooled together to form one pool. Thus, there were 3 pools per group with each pool containing 3 hippocampi. Hippocampal proteins 50 μg of γ-oryzanol, control, and standard (25 μg of γ-oryzanol and 25 μg of control) samples were labeled with CyDye DIGE Fluor minimal dyes (GE Healthcare). The samples were pooled and precipitated with tricarboxylic acid cycle (TCA). The final pellet was resuspended in rehydration buffer (8 M urea, 2 M thiourea, 20 mM dithiothreitol (DTT), 2% (w/v) CHAPS, 0.5% (v/v) PharmalyteTM 3-11 NL, bromophenol blue) and loaded on 18 cm, pH 3–11 non-linear, Immobiline^TM^ Dry Strip (GE Healthcare) in an IPGphor isoelectric focusing system (GE Healthcare). Simultaneously, 200 μg of hippocampal protein sample was precipitated with TCA and dissolved in the rehydration buffer. The sample was loaded on 18 cm, pH 3–11 non-linear, ImmobilineTM Dry Strip in an IPGphor isoelectric focusing system, and the protein spots were stained by Coomassie blue G-250 and excised for mass spectrometry analysis. After 12 h of active rehydration (30 V), isoelectric focusing was performed stepwise for 1 h at 200 V, 500 V and 1000 V respectively, then slowly increased to 8000 V for 2.5 h and finally decreased to 500 V for 2.5 h. The focused isoelectric focusing strips were equilibrated in equilibration buffer (6 M urea, 50% (v/v) glycerol, 1.5 mM Tris/HCl pH 8.8, 1% (w/v) SDS) containing 1% (w/v) DTT for 15 min and then 2.5% (w/v) iodacetamide for 15 min in the dark. Sodium dodecyl sulfate–polyacrylamide gel electrophoresis (SDS-PAGE) was performed on 11% polyacrylamide gels to separate the proteins at 60 V per gel for 2 h followed by 100 V per gel overnight at 20 °C using an EttanTM DALTsix system (GE Healthcare). Gels were then scanned by a fluorescence scanning, Fluoro Image Analyzer FLA-5100 (Fujifilm) and analyzed proteins spots by the Delta2D software (DECODON). 

### 2.8. Peptide Preparation

For protein identification gel pieces were subjected to in-gel digestion [31,32]. In brief, slices were washed consecutively with water, 50% acetonitrile (ACN), and 100% ACN. Proteins were reduced with 20 mM DTT in 50 mM ammonium bicarbonate and alkylated with 40 mM acryl amide (in 50 mM ammonium bicarbonate) for 30 min at room temperature. The slices were washed again and dehydrated with ACN. Dried slices were incubated with 330 ng trypsin (sequencing grade Promega, Mannheim, Germany) at 37°C overnight. The peptide extract was separated and remaining peptides extracted with 50% ACN. Peptides were dried in a vacuum concentrator and stored at –20°C.

### 2.9. Liquid Chromatography–Mass Spectrometry (LC–MS) analysis

Peptides were re-dissolved in 10 µL 0.1% formic acid. 3 µL were injected onto a C18 trap column (20 mm length, 100 µm inner diameter) coupled to a C18 analytical column (200 mm length, 75 µm inner diameter), made in house with 1.9 µm ReproSil-Pur 120 C18-AQ particles (Dr. Maisch, Ammerbuch, Germany). Solvent A was 0.1% formic acid. Peptides were separated during a linear gradient from 1% to 35% solvent B (90% ACN, 0.1% formic acid) within 15 min at a flow rate of 300 nL/min. The nano-high-performance liquid chromatography (HPLC) was coupled online to an LTQ Orbitrap Velos mass spectrometer (Thermo Fisher Scientific, Bremen, Germany). Peptide ions between 330 and 1600 m/z were scanned in the Orbitrap detector with a resolution of 30,000. The 25 most intense precursor ions (threshold intensity 5000) were subjected to collision induced dissociation and fragments analyzed in the linear ion trap. Fragmented peptide ions were excluded from repeat analysis for 10 s. Raw data processing and analysis of database searches were performed with Proteome Discoverer software 2.2.0.388 (Thermo Fisher Scientific). Peptide identification was done with an in house Mascot server version 2.6.1 (Matrix Science Ltd, London, UK). MS2 data were searched against Mus musculus sequences from SwissProt (release 2018_07). Precursor Ion m/z tolerance was 10 ppm, fragment ion tolerance 0.5 Da. Tryptic peptides were searched with up to two missed cleavages. Low-scoring spectrum matches were searched again with semitryptic specificity with up to one missed cleavage. Propionamide was set as a static modification (Cys). Oxidation (Met) and acetylation (protein N-terminus) were set as dynamic modifications. Mascot results from searches against SwissProt were sent to the percolator algorithm [33] version 3.00 as implemented in Proteome Discoverer. Only proteins with two peptides (maximum q-value 1%) were considered identified. Label-free quantification was based on unique peptide intensities. 

### 2.10. Western Blot Analysis

After 2D-DIGE, the same samples of mice hippocampi were utilized to perform western blot analysis. Hippocampal proteins 20 μg of γ-oryzanol and control were electrophoresed in 12% acrylamide gel and electro-blotted onto nitrocellulose membranes (Sigma-Aldrich, Merck KGaA, Darmastadt, Germany). Membranes were blocked for 1 h in 5% w/v Bovine Serum Albumin in TBS-T (0.1 M Tris-HCl pH 7.4, 0.15 M NaCl, 0.1% Tween 20) and incubated overnight at 4 °C with primary antibodies. Primary antibodies were anti-glyceraldehyde-3-phosphate dehydrogenase (GAPDH) (1:2500, Sigma-Aldrich), anti-α-synuclein (1:1000, BD Biosciences), anti-glial fibrillary acidic protein (1:500, Sigma-Aldrich), anti-apolipoprotein E (1:1000, Abcam), anti-cytochrome b-c1 complex subunit Rieske (1:1000, Abcam), and anti-glutathione S-transferase μ1 (1:1000, Proteintech). IR Dye near-infrared dyes-conjugated secondary antibodies (LI-COR, Lincoln, NE, USA) were used. The immunodetection was performed using a dual-mode western imaging system Odyssey FC (LI-COR, Lincoln, NE, USA). Quantification was performed using Image Studio Software (LI-COR, Lincoln, Nebraska, USA) and the results were normalized over the GAPDH signal.

### 2.11. Statistical Analysis

Two-way repeated measures analysis of variance (ANOVA) with Bonferroni post-test was used to determine the significance of the biological effect on the exploration of familiar and novel objects (NOR). An unpaired t-test with Welch’s correction was used to evaluate the discrimination index of NOR, parameters of Y-maze, and rotarod tests and western blot analysis of γ-oryzanol treatment versus control. Statistical analysis of protein expression (2D-DIGE) from γ-oryzanol compared with control were obtained by the Delta2D software (DECODON) using Student’s t-test, and the average of each protein spot intensity was normalized. Data are presented as means ± standard error of the mean (SEM), all statistical analyses were performed using Graph Pad Prism version 6 (Graph Pad Software, San Diego, CA, USA) and the statistical significance level set at *p* < 0.05.

## 3. Results

### 3.1. γ-Oryzanol Improves Cognitive Function in Adult Mice

**Figure 1:**
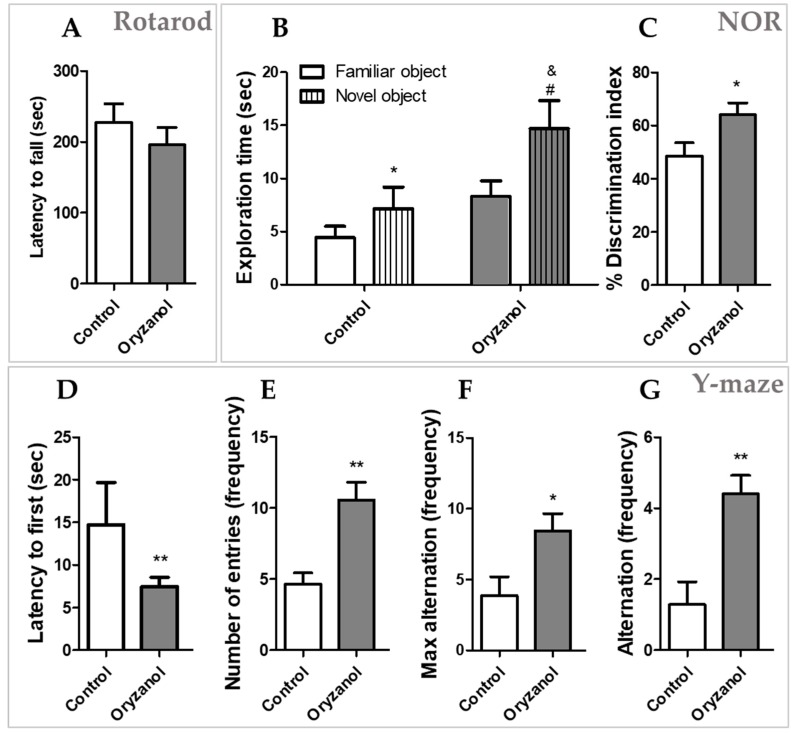
Behavioral tests of mice treated with γ-oryzanol or vehicle (control). After 21-day treatment with γ-oryzanol (*n* = 10) or vehicle (*n* = 10), mice were subjected to rotarod (**A**), novel object recognition (NOR) (**B**,**C**), and Y-maze (**D**–**G**) tests. Rotarod task measure the time spent on the top of the rotarod treadmill to evaluate motor activity (**A**). The NOR task assessed the time spent to explore the familiar and novel objects (**B**) and discrimination index (**C**). Different tasks were considered for Y-maze test: (**D**) time (sec) latency to entry first in the arm; (**E**) the number of arm entries; (**F**) max alternation, defined as the number of possible alternations counted by the total number of arms entered minus 2; (**G**) alternation frequency, defined as consecutive entries into 3 different arms and counted only if a mouse entered into the 3 arms of maze (without revisiting the first arm at the third visit). Data are shown as mean ± standard error of the mean (SEM). For Y-maze: * *p* < 0.05 and ** *p* < 0.005 vs. control; for NOR: * *p* < 0.05 vs. familiar object of control, # *p* < 0.05 vs. familiar object of γ-oryzanol, and & *p* < 0.05 vs. novel object of control.

Two groups of mice were administered daily with γ-oryzanol 100 mg/kg (*n* = 10) or vehicle (control, *n* = 10) for 21 days. After the treatment, mice were subjected to sequentially behavioral battery tests: first rotarod for locomotor activity, then Y-maze for spatial memory, and NOR for long-term memory prior to sacrifice and tissue collection. As shown in Figure 1A, the rotarod test was first performed and showed no significant difference between mice treated with γ-oryzanol compared with vehicle (*p* = 0.39), suggesting that γ-oryzanol does not alter the mobility of the animals. In Y-maze test, mice treated with γ-oryzanol spent less time to enter in the arm of the Y-maze (Figure 1D, *p* < 0.05) and showed a higher number of entries in the arms (Figure 1E, *p* < 0.05) compared to control group. The parameters of max alternation (Figure 1F, *p* < 0.05) and alternation (Figure 1G, *p* < 0.05) were also higher in γ-oryzanol treatment compared with control group, suggesting that γ-oryzanol improves cognitive behavioral abilities to remember spatial location and exploration of maze in adult mice. NOR includes 3 phases: habituation (day 1), acquisition trial (day 2), and actual test (day 3). In the habituation phase, total distance traveled was similar in γ-oryzanol and control mice. Also in the acquisition trial, there was no significant difference between γ-oryzanol and control in the time spent to explore the 2 identical objects (data not shown). In the actual test (day 3) both γ-oryzanol and control mice spent more time to explore the novel object than familiar one (Figure 1B, *p* < 0.05). However, the interest for the novel object showed by γ-oryzanol-treated mice was significantly higher than that of vehicle-treated mice (Figure 1B, *p* < 0.05). Likewise, discrimination index analysis confirmed that γ-oryzanol-treated mice had a higher interest towards the novel object compared to control mice (Figure 1C *p* < 0.05), suggesting the improvement of long-term memory to recognize the familiar and novel objects in adult mice. 

### 3.2. γ-Oryzanol Modulates Hippocampal Proteome

**Figure 2:**
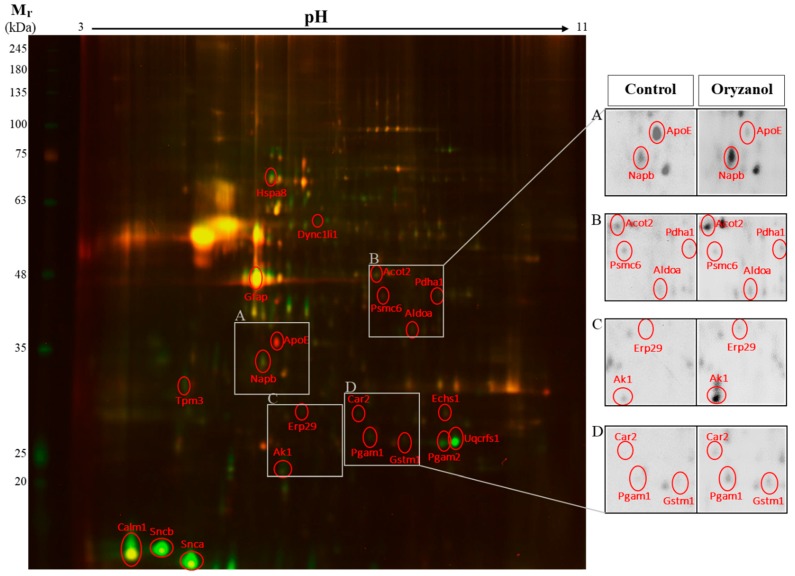
2D-difference gel electrophoresis (2D-DIGE) hippocampal proteome map of mice treated with γ-oryzanol or vehicle (control). 2D-DIGE proteome map shows the overlap between γ-oryzanol (green) and control (red) hippocampal proteome maps, and the overlapped spots are present in yellow. Mr: relative molecular mass. Some protein spots are shown enlarged in grey scales for better spot visualization (**A**–**D**).

**Figure 3:**
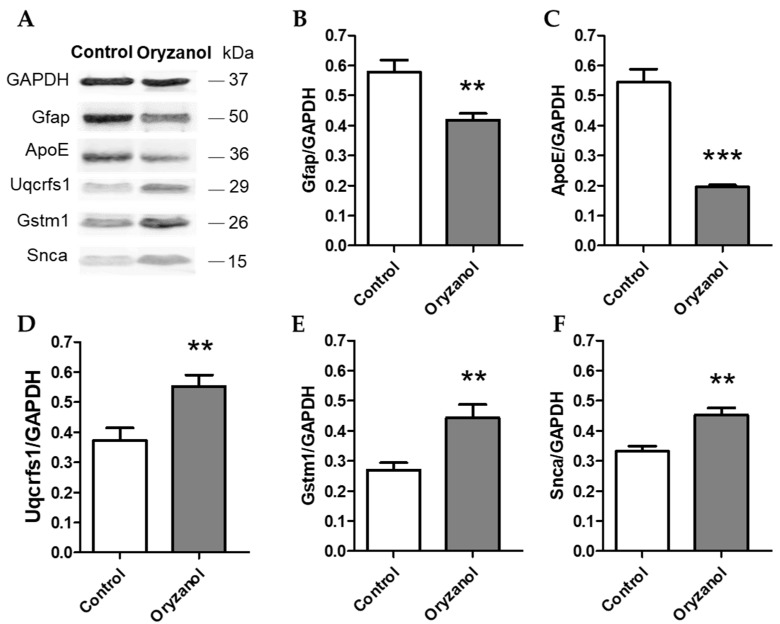
Western blot analysis of hippocampal proteome from mice treated with γ-oryzanol and vehicle (control). (**A**) Representative experiment of 12 % sodium dodecyl sulfate–polyacrylamide gel electrophoresis (SDS-PAGE) gel of γ-oryzanol and control blotted with different antibodies against glial fibrillary acidic protein (Gfap), apolipoprotein E (ApoE), cytochrome b-c1 complex subunit Rieske (Uqcrfs1), glutathione S-transferase μ1 (Gstm1), and α-synuclein (Snca). The immunodetection was performed using a dual-mode western imaging system Odyssey FC (LI-COR Lincoln, NE, USA). (**B**–**F**) Quantitative analysis of fluorescence signal of each group was performed using Image Studio Software (LI-COR, Lincoln, NE, USA) and the results were normalized over the GAPDH signal. Data are expressed as mean ± SEM; *** *p* < 0.001 and ** *p* < 0.01 vs. control.

To assess the quantitative changes of hippocampal protein expression between γ-oryzanol and control, 2D-DIGE was performed. 2D-DIGE is a valuable proteomics approach able to simultaneously separate 3 samples labeled with different fluorescent dyes on a single gel in order to eliminate inter-gel variations. The gels were scanned, and a total of 250–300 spots per gel were detected. The intensities of the protein spots were then quantified using the Delta2D software (DECODON). These spots were excised for in-gel trypsin digestion and analyzed by mass spectrometry for their identification. Figure 2 shows a representative 2D-DIGE proteome map in color scales, where γ-oryzanol and control samples were labeled with different CyDye DIGE Fluor minimal dyes. It displays the overlap between the γ-oryzanol (green) and control (red) hippocampal proteome maps, and the overlapped spots are present in yellow. Some protein spots are shown enlarged in grey scales for better spot visualization (insets, A–D). Based on the DECODON analysis performed on 4 different proteome maps, we identified 21 protein spots that significantly differed between γ-oryzanol and control groups and had the fold change, log2 of the ratio between γ-oryzanol and control, more than ± 0.3 (equal to ± 1.2 of the ratio between groups). Among the proteins identified, 18 proteins were significantly increased and 3 proteins were significantly decreased by γ-oryzanol compared with control. These proteins are classified by their function into 3 categories: synaptic plasticity and neuronal trafficking, neuroprotection and antioxidant activity, and mitochondria and energy metabolism (Table 1). Additionally, using western blot analysis we confirmed the significant increase of α-synuclein (Snca), glutathione S-transferase μ1 (Gstm1), and cytochrome b-c1 complex subunit Rieske (Uqcrfs1) in hippocampi of γ-oryzanol-treated mice compared with those of the control. By contrast, glial fibrillary acidic protein (Gfap) and apolipoprotein E (ApoE) were significantly decreased by γ-oryzanol compared with control, supporting the results observed in 2D-DIGE (Figure 3).

## 4. Discussion

Here, we demonstrated for the first time that long-term consumption of γ-oryzanol improves working and long-term memory in adult mice (12–14 months old) through regulating quantitative changes on hippocampal proteome. In particular, we found that mice treated with γ-oryzanol showed higher performance in Y-maze and NOR tests compared with vehicle-treated mice. Such behavior was accompanied by changing in the expression of hippocampal proteome involved in synaptic plasticity and neuronal trafficking, neuroprotection and antioxidant activity, and mitochondria and energy metabolism. In order to better clarify the relationship between the effects of γ-oryzanol on hippocampal proteome and its involvement in cognitive function, we categorized and discussed these proteins considering their function as follows. 

### 4.1. Synaptic Plasticity and Neuronal Trafficking

A significant increase of calmodulin 1 (Calm1), α-synuclein (Snca), β-synuclein (Sncb), β-soluble NSF attachment protein (Napb), tropomyosin α-3 chain (Tpm3), and cytoplasmic dynein 1 light intermediate chain 1 (Dync1li1) and significant decrease of 26S proteasome regulatory subunit 10B (Psmc6) were found in mice treated with γ-oryzanol compared to the control. All these proteins coordinate in a sequence of events to orchestrate synaptic plasticity and neuronal trafficking. 

In particular, Calm1 is a primary regulator of calcium (Ca^2+^) signaling pathways able to form Ca^2+^/calmodulin complex, leading to activation of Ca^2+^/calmodulin-dependent protein kinase II (CaMKII) [34,35]. This event is required in learning and memory processes [36,37,38,39]. For example, studies showed that CaMKII interacts with N-Methyl-D-aspartate (NMDA) receptors resulting in phosphorylation of CaMKII. This post-translational modification isoform promotes dendritic growth and synaptic formation, while the blockade of CaMKII activity with CaMKII inhibitors prevents these events [40,41]. Interestingly, a study of regulator of calmodulin signaling (RCS) knockout mice reported a decrease of explorative performance in elevated plus maze and open field tests compared with wild type mice, supporting the notion that this pathway is required in behavioral performance [42].

An important event in synaptic transmission is the synaptic vesicle (SV) exocytosis releasing neurotransmitters repeatedly, upon Ca2+ influx at the presynaptic neurons [43]. This cycle of the synaptic transmission requires continuous assembly and disassembly of soluble N-ethylmaleimide- sensitive fusion attachment protein receptor (SNARE) complexes [43,44,45]. Studies in synucleins knockout mice and other experimental models revealed that Snca plays a role in mediating SNARE-protein assembly, controlling SV exocytosis, and determining the presynaptic terminal size of neurons. These are related to the long-term organization of the nervous system [44,46,47]. In cells, deletion of Snca was found to lower the distal pool of presynaptic vesicles, and in mice, knockout of Snca also showed a decline in learning and working memory [48,49,50]. Napb is another cofactor of N-ethylmaleimide-sensitive factor (NSF) essential for SNARE-protein disassembly, SV exocytosis, and neurotransmission from presynaptic bouton [51,52,53]. These events are important for long-term potentiation (LTP) and synaptic plasticity in learning and memory consolidation [53,54,55]. 

Therefore, these findings support the hypothesis of γ-oryzanol as a nootropic compound in modulating neuronal maturation and retrograde transport (Tpm3 and Dync1li1) [56,57] and synaptic transmission and plasticity (Calm1, Psmc6 [58], Snca, Sncb, and Napb) thereby improving cognitive performance in adult mice.

### 4.2. Neuroprotection and Antioxidant Activity

γ-Oryzanol-treated mice showed increased expression of the heat shock cognate 71 kDa protein (Hspa8), the endoplasmic reticulum resident protein 29 (Erp29), and glutathione S-transferase μ1 (Gstm1) in the hippocampus, while glial fibrillary acidic protein (Gfap) was found decreased.

Hspa8 belongs to the heat shock protein 70 chaperone family possessing multiple neuroprotective effects [59]. It functions as a chaperone-mediated autophagic protein to maintain protein homeostasis and prevents neuronal death from metabolic stress and oxidative damage [60]. Hspa8 is also involved in mitochondrial protein import and β-amyloid (Aβ) clearance in the brain [61]. Erp29 is an endoplasmic reticulum (ER) luminal protein acting as a molecular chaperon in ER homeostasis, protein folding and trafficking, and cell survival [62]. A recent study indicated that Erp29 exerts neuroprotective effects through protecting neurons from apoptosis and promoting neuronal regeneration [63]. Gfap is the hallmark intermediate filament protein uniquely found in mature reactive astrocytes [64,65]. Studies have shown a positive relationship between Gfap levels and chronic inflammation and aging as well as neurodegenerative diseases, such as AD and Parkinson’s disease (PD) [66,67,68]. Lastly, glutathione-S-transferase (Gst) is a multifunctional antioxidant enzyme protecting the brain against toxics and oxidative stress, which are involved in neurodegenerative diseases [69,70]. Interestingly, Gst is a target of Nrf2 signaling pathway. We recently demonstrated that γ-oryzanol is able to activate Nrf2 pathway and its target genes in an in vitro model, resulting in enhancement of nucleophilic tone to maintain redox homeostasis [13,14]. Together, these data support our previous finding of γ-oryzanol as a modulator of Nrf2/Gst pathway and highlight γ-oryzanol as a neuroprotective compound in aging.

### 4.3. Mitochondria and Energy Metabolism

Glucose metabolism (glycolysis) and fatty acid metabolism (β-oxidation) are two of the main sources of energy in the brain [71,72]. Herein, we found that long-term treatment of γ-oryzanol increases the expression of fructose-bisphosphate aldolase A (Aldoa), phosphoglycerate mutase 1, 2 (Pgam1, 2), acyl-coenzyme A thioesterase 2 (Acot2), enoyl-CoA hydratase 1 (Echs1), pyruvate dehydrogenase 1 (Pdha1), cytochrome b-c1 complex subunit Rieske (Uqcrfs1), adenylate kinase isoenzyme 1 (Ak1), and carbonic anhydrase 2 (Car2) and decreases the expression of apolipoprotein E (ApoE). The fact that γ-oryzanol positively regulates the expression of all these proteins in the hippocampus suggests its beneficial effects in brain energy metabolism, facilitating energy to its consuming sites [73,74], and controlling neural pH [75,76] and the signaling network associated with learning and memory function. 

In particular, Aldoa and Pgam1, 2 are enzymes in the glycolytic pathway playing a role in maintenance of glucose homeostasis [77,78]. Acot2 and Echs1 belong to series of enzymes involved in catalyzing long-chain fatty acyl esters to acetyl-CoA [79,80]. Acetyl-CoA is also converted from pyruvate, the output of glycolysis, by pyruvate dehydrogenase [79,80]. Pdha1 is, therefore, a mitochondrial multi-enzyme acting as a rate-limiting step connecting glycolysis to the tricarboxylic acid (TCA) cycle and oxidative phosphorylation [81,82,83]. Uqcrfs1, as a member of the Rieske family, is a component of the mitochondrial electron transport chain (ETC)-complex III or cytochrome b-c1 complex [84,85]. The impairment of Uqcrfs1 leads to an excessive reactive oxygen species (ROS) and the dysfunction of energy homeostasis [85,86]. Numerous studies demonstrated that low expression of proteins such as Aldoa, Pdha1, and Car2 was found in neurodegenerative diseases including AD, PD, and Lewy Body [75,87,88,89]. 

ApoE is a multifunctional protein consisting of 3 major isoforms (ApoE2, ApoE3, and ApoE4) with the central role in lipid and neuronal homeostasis [90]. In the brain, ApoE is produced mainly by astrocytes and expressed in neurons responsive to excitotoxic injury [90,91]. ApoE has been found to be involved in neurotoxicity, mitochondrial dysfunction, and neurodegenerative diseases, particularly AD [92]. Studies in a mice model demonstrated that ApoE is required for amyloid plaque formation [93], and lower ApoE expression is associated with the decrease of Aβ related pathological changes in the cortex and hippocampus [94,95,96]. Furthermore, higher levels of ApoE in the brain were able to induce abnormally phosphorylated tau protein resembling pre-neurofibrillary tangles and also cognitive impairment in mice [97,98]. Likewise, an augmentation of CSF ApoE levels was also related to higher CSF tau levels and cognitive decline in human [99]. Therefore, the decrease of ApoE expression by γ-oryzanol treatment in adult mice might be beneficial in the global context of neuronal welfare and functions in aging.

## 5. Conclusions

Overall, these findings suggested γ-oryzanol as a natural compound able to maintain and reinforce brain function. Such effects might allow the brain to better respond to noxious stimuli occurred during the entire life and prevent or postpone the development of neurodegenerative diseases. 

Although more intensive studies are needed, we propose γ-oryzanol as a putative dietary phytochemical for preserving brain reserve, the ability to tolerate age-related changes, thereby preventing clinical symptoms or signs of neurodegenerative diseases.

## Figures and Tables

**Table 1:** List of the proteins modulated by γ-oryzanol.

Accession No.	Description	Coverage [%]	MW [kDa]	pI	*p* Value	** Fold Change
**Synaptic Plasticity and Neuronal Trafficking**						
Q91ZZ3	β-Synuclein (Sncb)	43	14.0	4.37	0.005	2.160
O55042	α-Synuclein (Snca)	82	14.5	4.77	0.005	1.874
P0DP26	Calmodulin-1 (Calm1)	52	16.8	4.22	0.003	0.799
P21107	Tropomyosin α-3 chain (Tpm3)	48	33.0	4.72	0.017	1.033
P28663	Beta-soluble NSF attachment protein (Napb)	77	33.5	5.47	0.008	0.357
P62334	26S proteasome regulatory subunit 10B (Psmc6)	42	44.1	7.49	0.039	−0.421
Q8R1Q8	Cytoplasmic dynein 1 light intermediate chain 1 (Dync1li1)	53	56.6	6.42	0.028	1.098
**Neuroprotection and antioxidant activity**						
P10649	Glutathione S-transferase μ1 (Gstm1)	59	26.0	7.94	0.009	0.677
P57759	Endoplasmic reticulum resident protein 29 (Erp29)	36	28.8	6.15	0.039	0.694
P03995	Glial fibrillary acidic protein (Gfap)	30	49.9	5.34	0.013	−0.408
P63017	Heat shock cognate 71 kDa protein (Hspa8)	52	70.8	5.52	0.007	0.319
**Mitochondria and energy metabolism**						
Q9R0Y5	Adenylate kinase isoenzyme 1 (Ak1)	47	21.5	5.81	0.000	1.308
Q9DBJ1	Phosphoglycerate mutase 1 (Pgam1)	80	28.8	7.18	0.000	1.463
O70250	Phosphoglycerate mutase 2 (Pgam2)	15	28.8	8.50	0.000	1.401
P00920	Carbonic anhydrase 2 (Car2)	42	29.0	7.01	0.001	1.539
Q9CR68	Cytochrome b-c1 complex subunit Rieske, mitochondrial (Uqcrfs1)	19	29.3	8.70	0.000	2.014
Q8BH95	Enoyl-CoA hydratase, mitochondrial (Echs1)	30	31.5	8.48	0.001	1.226
P08226	Apolipoprotein E (ApoE)	19	35.8	5.68	0.000	−2.826
P05064	Fructose-bisphosphate aldolase A (Aldoa)	61	39.3	8.09	0.001	0.413
P35486	Pyruvate dehydrogenase E1 component subunit α, somatic form, mitochondrial (Pdha1)	38	43.2	8.19	0.046	0.348
Q9QYR9	Acyl-coenzyme A thioesterase 2, mitochondrial (Acot2)	35	49.6	7.36	0.048	0.548
** **Fold change** = log2 [ratio (γ-oryzanol/control)]						

The table reports quantitative changes of proteins with coverage (%), isoelectric point (pI), molecular weight (MW), p value, and fold change (log2 of the ratio between γ-oryzanol and control). The proteins are categorized by their function into 3 groups: synaptic plasticity and neuronal trafficking, neuroprotection and antioxidant activity, and mitochondria and energy metabolism.
